# CAPS1 stabilizes the state of readily releasable synaptic vesicles to fusion competence at CA3–CA1 synapses in adult hippocampus

**DOI:** 10.1038/srep31540

**Published:** 2016-08-22

**Authors:** Yo Shinoda, Chiaki Ishii, Yugo Fukazawa, Tetsushi Sadakata, Yuki Ishii, Yoshitake Sano, Takuji Iwasato, Shigeyoshi Itohara, Teiichi Furuichi

**Affiliations:** 1 Department of Applied Biological Science, Tokyo University of Science, Noda, Chiba 278-8510, Japan; 2 School of Pharmacy, Tokyo University of Pharmacy and Life Sciences, Hachioji, Tokyo 192-0392, Japan; 3 Department of Brain Structure and Function, Faculty of Medical Sciences, University of Fukui, Yoshida-gun, Fukui 910-1193, Japan; 4 Advanced Scientific Research Leaders Development Unit, Gunma University, Maebashi, Gunma 371-8511, Japan; 5 Division of Neurogenetics, National Institute of Genetics, Mishima, Shizuoka 411-8540, Japan; 6 Department of Genetics, SOKENDAI, Mishima, Shizuoka 411-8540, Japan; 7 Laboratory for Behavioral Genetics, RIKEN Brain Science Institute, Wako, Saitama 351-0198, Japan

## Abstract

Calcium-dependent activator protein for secretion 1 (CAPS1) regulates exocytosis of dense-core vesicles in neuroendocrine cells and of synaptic vesicles in neurons. However, the synaptic function of CAPS1 in the mature brain is unclear because *Caps1* knockout (KO) results in neonatal death. Here, using forebrain-specific *Caps1* conditional KO (cKO) mice, we demonstrate, for the first time, a critical role of CAPS1 in adult synapses. The amplitude of synaptic transmission at CA3–CA1 synapses was strongly reduced, and paired-pulse facilitation was significantly increased, in acute hippocampal slices from cKO mice compared with control mice, suggesting a perturbation in presynaptic function. Morphological analysis revealed an accumulation of synaptic vesicles in the presynapse without any overall morphological change. Interestingly, however, the percentage of docked vesicles was markedly decreased in the *Caps1* cKO. Taken together, our findings suggest that CAPS1 stabilizes the state of readily releasable synaptic vesicles, thereby enhancing neurotransmitter release at hippocampal synapses.

A critical event in synaptic transmission is the exocytosis of synaptic vesicles (SVs), which is carried out by the fusion machinery consisting of the SNARE protein complex and various associated proteins^1^. Docking at the presynaptic active zone and priming to fusion competence are critical steps leading up to the fusion of SVs with the plasma membrane^2^,^3^. The molecular mechanisms underlying the docking and priming steps are tightly regulated and control not only basal synaptic transmission but also synaptic efficacy, and are major contributors to synaptic plasticity, which is the cellular basis of learning and memory^4^. However, the mechanisms controlling the docking and priming steps of SV fusion are not fully understood.

The calcium-dependent activator protein for secretion (CAPS) family consists of two distinct isoforms, CAPS1 and CAPS2, which play a role in the secretion of dense-core vesicles (DCVs)^5^,^6^,^7^,^8^,^9^,^10^,^11^,^12^. Expression of CAPS1 and CAPS2 is widespread in the mouse brain and is complementary in many brain regions^13^. CAPS2 promotes the secretion of brain-derived neurotrophic factor (BDNF), likely via the release of DCV-like secretory granules, in cultured cerebellar granule cells^9^, cerebral cortical neurons^14^ and hippocampal neurons^15^,^16^. CAPS2 regulates BDNF release kinetics, including frequency and amplitude^15^. A role for CAPS2 in synaptic transmission *in vivo* has been shown by studies in *Caps2* knockout (KO) mice, which revealed changes in the paired-pulse ratio (PPR), but no differences in excitatory post-synaptic potential (EPSP), in parallel fibre–Purkinje cell synapses in the cerebellum^17^, and no detectable alteration of EPSP in hippocampal synapses^15^,^18^. CAPS1 has been shown to regulate the exocytosis of DCVs in adrenal chromaffin cells, pancreatic β cells and PC12 cells^10^,^11^,^19^,^20^,^21^. CAPS1 has a domain that is homologous to Munc13 (a priming factor)^6^,^22^, to which syntaxin-1 (one of two t-SNARE proteins on the plasma membrane) binds^23^,^24^,^25^. The interaction of CAPS1 and/or Munc13-1 with syntaxin-1 has been suggested to induce fusion competence (priming) of DCVs^10^,^12^,^21^,^26^ and SVs^12^,^18^. Although there are studies using *in vitro* microisland cultures and organotypic cultures from E18–P0 *Caps1* KO mouse pups^18^,^27^, it remains unclear whether CAPS1 regulates SV release in the adult brain because *Caps1* KO mice die soon after birth.

In this study, we examined the role of CAPS1 in the exocytosis of SVs using forebrain-specific *Caps1* conditional KO (cKO) mice that are able to mature to adulthood^28^ (Supplemental Fig. S1). Our results show that CAPS1 deficiency decreases activity-dependent SV release events *in vivo* at CA3–CA1 synapses in adult hippocampal slices. In addition, it causes the accumulation of SVs near the active zone but reduces the number of SVs at the plasma membrane of presynaptic terminals. Collectively, our results for the first time indicate that CAPS1 stabilizes the state of readily-releasable SVs at mature synapses in the adult hippocampus.

## Results

### *Caps1* cKO reduces the release probability at CA3–CA1 synapses in acute hippocampal slices

**Figure 1:**
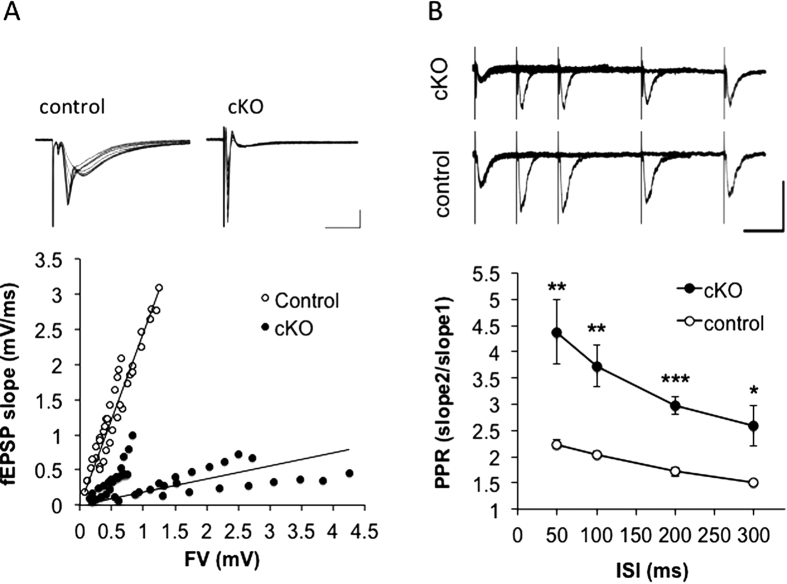
Presynaptic reduction in basal synaptic transmission in *Caps1* cKO hippocampus. (**A**) Input-output curve for CA3–CA1 synapses in control (*Caps1*^*flox/*−^*/Emx1*^*wt/wt*^) and *Caps1* cKO (*Caps1*^*flox/***−**^*/Emx1*^*Cre/wt*^) hippocampal slices. Representative traces are provided above (scale bars: 10 ms, 1 mV). The graph below shows fEPSP slopes as a function of fibre volley (FV). The factors of proportionality are 2.4425 for control and 0.1856 for *Caps1* cKO. *P* < 0.001, analysis of covariance (ANCOVA); n = 5 animals each for control and *Caps1* cKO. (**B**) Paired-pulse ratio (PPR) at CA3–CA1 synapses in control and *Caps1* cKO hippocampal slices. Representative traces are provided above (scale bars: 50 ms, 1 mV). The graph below shows PPR as a function of ISI. *P < 0.05, **P < 0.01, ***P < 0.001, Student’s *t*-test; n = 6 animals for control and n = 5 animals for *Caps1* cKO.

To clarify whether CAPS1 is involved in the exocytosis of SVs *in vivo* in the adult brain, we prepared acute hippocampal slices from *Caps1* cKO mice and their control littermates at postnatal 8 weeks and recorded basal synaptic transmission at CA3–CA1 synapses (Fig. 1A). Input-output curves were constructed using the amplitude of fibre volley and slope of field EPSP (fEPSP) for each electrical stimulus. Slices from *Caps1* cKO animals showed a significant reduction in fEPSP compared with control slices (factors of proportionality: control, 2.4425 [n = 5]; *Caps1* cKO, 0.1856 [n = 5]; analysis of covariance, *P* < 0.001). To evaluate the presynaptic and postsynaptic contributions to this reduction in fEPSP, we measured paired-pulse facilitation (PPF) at CA3–CA1 synapses (Fig. 1B). PPFs at every interstimulus interval (ISI) tested (50, 100, 200 and 300 ms) were dramatically increased in *Caps1* cKO slices compared with control (50 ms: PPR control = 2.24 ± 0.07, PPR *Caps1* cKO = 4.38 ± 0.61, *P* < 0.01; 100 ms: PPR control = 2.05 ± 0.06, PPR *Caps1* cKO = 3.73 ± 0.40, *P* < 0.01; 200 ms: PPR control = 1.72 ± 0.08, PPR *Caps1* cKO = 2.98 ± 0.17, *P* < 0.001; 300 ms: PPR control = 1.51 ± 0.05, PPR *Caps1* cKO = 2.58 ± 0.38, *P* < 0.05; Student’s *t*-test, n = 6 and 5 for control and *Caps1* cKO, respectively). These results demonstrate, for the first time, that CAPS1 deficiency reduces basal synaptic transmission, at least in part, by diminishing the release probability of SVs at CA3–CA1 synapses in acute adult hippocampal slices.

### *Caps1* cKO causes aberrant accumulation of SVs at CA3–CA1 synapses

**Figure 2:**
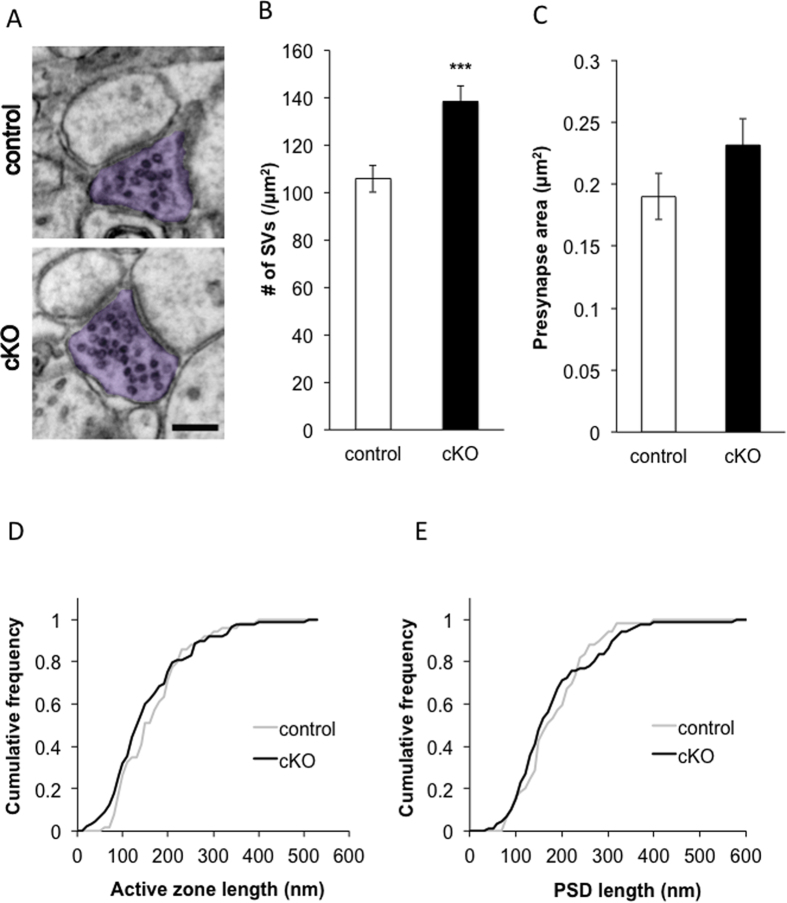
SV accumulation in the *Caps1* cKO presynapse. (**A**) Representative images of presynaptic boutons in the CA3–CA1 stratum radiatum in control and *Caps1* cKO mice. The presynaptic bouton is coloured in purple (scale bar: 200 nm). (**B**) Number of SVs in control and *Caps1* cKO synapses; n = 48 and 51 synapses for control and *Caps1* cKO, respectively. ***P < 0.001, Student’s *t*-test. (**C**) The area of the presynapse in control and *Caps1* cKO synapses; n = 48 and 51 synapses for control and *Caps1* cKO, respectively. *P* = 0.14, Student’s *t*-test. (**D**) Cumulative frequency of active zone length in control and *Caps1* cKO synapses; n = 49 and 88 active zones for control and *Caps1* cKO, respectively. *P* = 0.33, Kolmogorov–Smirnov test. (**E**) Cumulative frequency of PSD length in control and *Caps1* cKO synapses; n = 49 and 86 PSDs for control and *Caps1* cKO, respectively. *P* = 0.61, Kolmogorov–Smirnov test.

Because our results suggested that CAPS1 deficiency reduces presynaptic release probability, we examined synaptic ultrastructure in the CA1 stratum radiatum of *Caps1* cKO mice by transmission electron microscopy (TEM) (Fig. 2A). The number of SVs per presynapse was significantly increased in *Caps1* cKO compared with WT control (Fig. 2B) (control: 105.7 ± 5.8 per μm^2^ [n = 48]; *Caps1* cKO: 138.5 ± 6.5 per μm^2^ [n = 51]; Student’s *t*-test, *P* < 0.001). However, the presynaptic area did not differ between control and *Caps1* cKO mice (Fig. 2C) (control: 0.19 ± 0.02 per μm^2^ [n = 48]; *Caps1* cKO: 0.23 ± 0.02 per μm^2^ [n = 51]; Student’s *t*-test, *P* = 0.14). Furthermore, there were no differences in the length of the active zone or postsynaptic density (PSD) between the two genotypes (active zone length: control [n = 49] vs. cKO [n = 88], Kolmogorov–Smirnov test, *P* = 0.33 [Fig. 2D]; PSD length: control [n = 49] vs. cKO [n = 86], Kolmogorov–Smirnov test, *P* = 0.61 [Fig. 2E]). Collectively, these results suggest that *Caps1* cKO causes the aberrant presynaptic accumulation of SVs at hippocampal CA3–CA1 synapses without significantly impacting the morphology of the presynaptic region, the active zone or the PSD.

### *Caps1* cKO perturbs the presynaptic distribution of SVs close to the active zone

**Figure 3:**
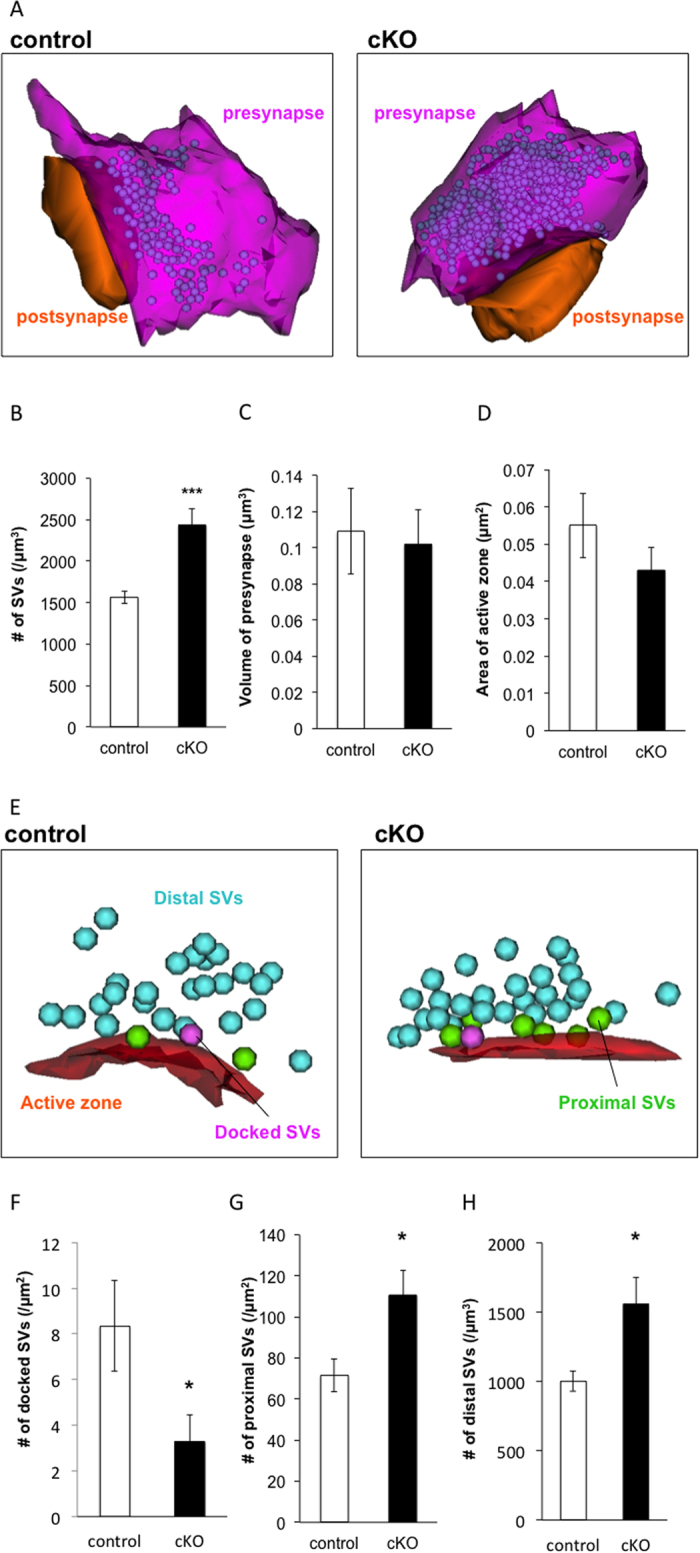
Docked vesicles are decreased at CA3–CA1 stratum radiatum synapses in *Caps1* cKO mice. (**A**) Representative 3D reconstructed image of synapses in control and *Caps1* cKO mice. Presynapse, postsynapse and SVs are coloured in magenta, orange and purple, respectively. (**B**) Number of SVs in control and *Caps1* cKO synapses. There is a significant accumulation of SVs in *Caps1* cKO animals; n = 17 and 19 synapses for control and *Caps1* cKO, respectively. ***P < 0.001, Student’s *t*-test. (**C**) Presynaptic volume in control and *Caps1* cKO mice; n = 17 and 19 synapses for control and *Caps1* cKO, respectively. *P* = 0.79, Student’s *t*-test. (**D**) Area of the active zone in control and *Caps1* cKO synapses; n = 19 and 22 active zones for control and *Caps1* cKO, respectively. *P* = 0.26, Student’s *t*-test. (**E**) Representative 3D reconstructed images of SVs around the active zone in control and *Caps1* cKO synapses. The active zone is colored orange. Docked, proximal and other SVs are colored magenta, green and pale blue, respectively. (**F**) Number of docked SVs; n = 19 and 22 active zones for control and *Caps1* cKO, respectively. *P < 0.05, Student’s *t*-test. (**G**) Number of proximal SVs; n = 19 and 22 active zones for control and *Caps1* cKO, respectively. *P < 0.05, Student’s *t*-test. (**H**) Number of distal SVs; n = 17 and 19 synapses for control and *Caps1* cKO, respectively. *P < 0.05, Student’s *t*-test.

To evaluate the distribution of SVs in the presynaptic region in greater detail, we performed three-dimensional scanning electron microscopy (3D-SEM) (Fig. 3A). 3D reconstruction of the synaptic ultrastructures showed that the number of SVs per volume of presynapse was significantly increased in *Caps1* cKO mice compared with control mice (control: 1 564.1 ± 189.5 per μm^3^ [n = 17]; *Caps1* cKO: 2 436.4 ± 130.7 per μm^3^ [n = 19]; Student’s *t*-test, *P* < 0.001) (Fig. 3B). The volume of the presynaptic bouton did not differ between the two genotypes (control: 0.11 ± 0.02 μm^3^ [n = 17]; *Caps1* cKO: 0.10 ± 0.02 μm^3^ [n = 19]; Student’s *t*-test, *P* = 0.79) (Fig. 3C). In addition, the area (μm^2^) of the active zone did not differ between control and *Caps1* cKO mice (control: 0.055 ± 0.009 μm^2^ [n = 19]; *Caps1* cKO: 0.043 ± 0.006 μm^2^ [n = 22]; Student’s *t*-test, *P* = 0.26) (Fig. 3D), as revealed by 2D-TEM analysis (Fig. 2). To facilitate analysis, we classified the SVs into one of three types according to distance from the active zone: (1) docked type, SVs attached to the membrane; (2) proximal type, SVs distributed within 50 nm of the active zone; and (3) distal type, SVs distributed greater than 50 nm from the active zone (Fig. 3E). The number of docked SVs was markedly reduced in *Caps1* cKO synapses compared with control synapses (control: 8.4 ± 2.0 per μm^2^ [n = 19]; *Caps1* cKO: 3.3 ± 1.2 per μm^2^ [n = 22]; Student’s *t-*test, *P* < 0.05) (Fig. 3F). Interestingly, the number of proximal SVs was significantly increased in *Caps1* cKO synapses compared with control synapses (control: 71.5 ± 7.9 per μm^2^ [n = 15]; *Caps1* cKO: 110.6 ± 12.2 per μm^2^ [n = 22]; Student’s *t*-test, *P* < 0.05) (Fig. 3G). In addition, the number of distal SV was significantly increased in *Caps1* cKO synapses compared with control synapses (control: 1,003.7 ± 74.3 per μm^3^ [n = 10]; *Caps1* cKO: 1,558.7 ± 189.2 per μm^3^ [n = 17]; Student’s *t*-test, *P* < 0.05) (Fig. 3H). Collectively, these results suggest that the acute loss of CAPS1 impairs the ability of presynaptic SVs to localize to or access the active zone, although it does not affect presynaptic volume or active zone area. The lack of CAPS1 therefore impacts the fusion competence of glutamatergic SVs at CA3–CA1 synapses.

## Discussion

Neurotransmitter release plays a key role in cognitive processes and behaviour, and is tightly regulated across the sequential stages of SV exocytosis. Although the SNARE fusion machinery and numerous associated proteins regulate exocytosis^29^, the underlying molecular mechanisms are not fully understood. In the present study, using forebrain-specific *Caps1* cKO mice and acute *Caps1* deletion in primary neurons, we demonstrate that CAPS1 is required for the proper exocytosis of SVs at CA3–CA1 synapses in the adult hippocampus.

A previous study showed a reduction in EPSC amplitude in primary hippocampal microisland cultures prepared from *Caps1*/*2* double KO (DKO) mice^18^. In the present study, stimulus-induced fEPSPs were significantly reduced in mature CA3–CA1 synapses in acute hippocampal slices prepared from adult *Caps1* cKO mice, suggesting that CAPS1 is involved in basal synaptic transmission in the mature hippocampus. Although a previous report using hippocampal microisland cultures revealed paired-pulse depression in autaptic synapses^18^, PPR was significantly increased in *Caps1*/*2* DKO neurons compared with control neurons^18^. This latter observation is in agreement with our present finding of a robust enhancement of PPF at mature CA3–CA1 synapses in *Caps1* cKO mice, to roughly twice that in control animals for ISIs of 50–300 ms. Thus, our findings suggest that CAPS1 regulates the release probability of SVs in mature hippocampal synapses in adult mice. Furthermore, time-lapse fluorescence imaging of recycling pool vesicles in neurons with acute *Caps1* deletion showed a significant reduction in the exocytosis of SVs (Supplemental Fig. S2). Collectively, these results show that the impaired synaptic release observed in the cKO is caused by the loss of CAPS1 function, rather than the result of an indirect developmental effect. Therefore, our findings suggest that CAPS1 regulates not only the secretion of DCVs containing neuropeptides, peptide hormones and monoamines^7^,^8^,^10^,^11^,^19^,^20^,^21^,^26^, but also the release of glutamate via SV exocytosis at CA3–CA1 synapses in the adult hippocampus, as *in vitro* experiment reported previously^18^.

Several presynaptic changes may underlie the reduction in fEPSP, including shrinkage of presynaptic boutons^30^, reduction in active zone size^31^, decrease in SV number^31^, reduction of Ca^2+^ sensitivity^32^, and reduction in SV release probability^33^ (resulting from a decrease in readily releasable vesicle pool size^34^ and/or the number of docked vesicles^35^). Ultrastructural analysis of CA3–CA1 synapses using 2D-TEM and 3D-SEM revealed no overall changes in presynaptic area, volume, active zone length and area in adult *Caps1* cKO animals. The total number of SVs was significantly increased; however, docked SVs were markedly decreased in *Caps1* cKO mice. Interestingly, proximal SVs, the SVs within 50 nm of the active zone, were significantly increased in the *Caps1* cKO. This suggests that the distribution of SVs is regulated, at least in part, by CAPS1.

Our ultra-structural studies of KO synapses showed a decrease in number of “docked vesicles”, defined as vesicles attached to the presynaptic membrane, but an increase in number of “undocked vesicles”, defined distal and proximal to the presynaptic membrane. Within “docked vesicles” we defined we could not distinguish between the “docking” and “priming” steps of synaptic vesicles during exocytosis event. However, our electrophysiological studies of KO synapses revealed a decrease in release probability compared to WT, suggesting a decrease in fusion-competent vesicles. Taken together, we suggest that CAPS1 is crucial for at least fusion competent and/or readily releasable vesicles that are distributed on the presynaptic membrane. Therefore, a CAPS1 deficiency may cause the accumulation of SVs in the presynaptic terminals of CAPS1 cKO mice. Similar morphological findings, especially for docked and proximal SVs, were reported by Imig and colleagues using organotypic slice cultures prepared from E18 *Caps1*/*2* DKO mice^27^, although the remarkable accumulation of SVs at presynaptic terminals in *Caps1* cKO cells observed in the present study was not detected in the culture system in their study^27^. This disparity may be caused by differences between acutely prepared slices and organotypic slice cultures^36^. Indeed, although docked vesicles may be severely reduced in *Caps1*/*2* DKO organotypic slice cultures^27^, morphological and electrophysiological properties can be influenced by the culture environment^36^. In the present study, release probability and magnitude were reduced in *Caps1* cKO mature hippocampal synapses, suggesting that SVs might accumulate in presynaptic terminals because of the loss of CAPS1.

CAPS1 has a domain that is homologous with the Munc13 family of proteins, suggesting that it may function as a priming factor for SV membrane fusion^37^. Indeed, mice lacking Munc13^3^ and RIM1^38^ (another presynaptic protein that regulates Ca^2+^ -dependent SV release) also exhibit a reduction in release probability, although without SV accumulation in presynaptic terminals^27^. Thus, CAPS1 may prime SVs for exocytosis, independently or in concert with Munc13 and RIM1. CAPS1 may regulate not only SV exocytosis, but also SV endocytosis following exocytosis. CAPS1 interacts with plasma membrane phosphatidylinositol 4,5-bisphosphate (PIP_2_)^39^, which has been reported to be involved in vesicle endocytosis in addition to exocytosis^40^. Thus, CAPS1 may also have the ability to regulate vesicular endocytosis, which may in part account for the significant reduction of recycling SVs in *Caps1* acute KO synapses (Supplemental Fig. S2F). Further study is required to clarify the functions of CAPS1 in the exocytotic and endocytotic pathways.

In conclusion, our findings suggest that CAPS1 plays a critical role in synaptic transmission by regulating the recruitment and/or access of SVs to the active zone. Thus, CAPS1 is involved in the fusion competency of SVs. CAPS1 may also be involved in the trafficking of SVs in both the exocytotic and endocytotic pathways. Our study using the forebrain-specific *Caps1* cKO mouse suggests that CAPS1 is not essential for the release of glutamate, but enhances synaptic transmission at CA3–CA1 synapses in the hippocampus. Conventional CAPS1 KO mice die soon after birth, suggesting that CAPS1 may have numerous critical functions that are dependent on cell type and the specific cargo released.

## Methods

### Animals

All experimental protocols were evaluated and approved by the Regulation for Animal Research at Tokyo University of Science. All experiments were conducted in accordance with the Regulations for Animal Research at the Tokyo University Science. The generation of forebrain-specific *Caps1* cKO C57BL/6 mice has been described previously^28^. Briefly, *Caps1*^*flox/*−^ females were crossed with *Caps1* and *Emx1*-Cre heterozygote (*Caps1*^+/−^*/Emx1*^*Cre/wt*^) males to produce control (*Caps1*^*flox/*−^*/Emx1*^*wt/wt*^) and *Caps1* cKO (*Caps1*^*flox/*−^*/Emx1*^*Cre/wt*^) offspring.

### Hippocampal acute slice preparation

Hippocampal slices were prepared as described previously^15^. Under deep anaesthesia, 8-week-old *Caps1* cKO and control male mice were decapitated. The brains were removed rapidly and put into ice-chilled high-sucrose Ringer’s solution ([in mM] 234 sucrose, 2.5 KCl, 1.25 NaH_2_PO_4_, 10 MgSO_4_, 0.5 CaCl_2_, 26 NaHCO_3_, 11 D-glucose). Using a vibratome (Dosaka, Kyoto, Japan), hippocampi were cut into transverse sections (500 μm thick) in the same ice-chilled high-sucrose Ringer’s solution and maintained in artificial cerebrospinal fluid (ACSF; [in mM] 125 NaCl, 2.5 KCl, 1.25 NaH_2_PO_4_, 1 MgCl_2_, 2 CaCl_2_, 26 NaHCO_3_, 11 D-glucose) for at least 2 hours at room temperature. All solutions were constantly bubbled with 95% O_2_/5% CO_2_ mixed gas.

### Electrophysiology

Electrophysiological recordings were performed as described previously^15^. All electrophysiological recordings were performed in ACSF at 26 °C. ACSF was exchanged at a rate of 1 ml/min. A bipolar tungsten-stimulating electrode (WPI) was placed in the CA1 stratum radiatum region. fEPSPs were recorded from the CA1 stratum radiatum following 0.05-Hz test pulses. The recording electrode was set in a glass pipette (Harvard Apparatus) filled with ACSF. Electrical signals were amplified using a MultiClamp 700A (Molecular Devices) and digitized at 10 kHz and filtered at 2 KHz using a Digidata 1440 system with pCLAMP10 software (Molecular Devices).

### Electron microscopy

Electron microscopic analysis was performed as described previously^41^ with partial modification. Eight-week-old *Caps1* cKO and control mice were anaesthetized with CO_2_ gas and perfused with PBS (0.9% NaCl in 0.1 M phosphate buffer) and then with modified Karnovsky fixative (0.8% paraformaldehyde (PFA) [TAAB], 1.5% glutaraldehyde [Nacalai] in 0.15 M phosphate buffer) (the procedure was performed by investigators blinded to genotype). The brains were removed and post-fixed in 4% PFA in 0.1 M phosphate buffer at 4 °C overnight. The fixed brains were cut into coronal sections (100 µm thick) using a vibratome. The slices are placed in cacodylate buffer containing 2% OsO_4_ and 1.5% potassium ferrocyanide for 1 h at room temperature, followed by subsequent treatments with 1% thiocarbohydrazide solution for 20 min and the second osimium staining (2% aqueous OsO_4_) for 30 min both at room temperature. The slices were then placed in 2% aqueous uranyl acetate at 4 °C overnight. The slices were subsequently treated with lead aspartate solution (0.066 g of lead nitrate in 10 ml of 0.003 M aspartic acid, pH 5.5) at 60 °C for 30 min. The slices were dehydrated with a graded ethanol series (70, 90, 100 and 100%, 10 min each at 0 °C) and mounted with Durcupan/Araldite. Then, 40-nm serial sections were prepared using an UltracutT microtome (Leica). Images of the CA1 stratum radiatum were collected, using one animal each for control and *Caps1* cKO, on a JEOL 1400CX electron microscope (JEOL) with a 100-kV beam. The images were analyzed using Photoshop (Adobe) and ImageJ (NIH) software. 3D reconstruction was carried out with Reconstruct software (SynapseWeb).

### Statistical analysis

If not stated otherwise, data are expressed as mean ± SEM. Differences between data sets were assessed using two-tailed Student’s *t*-test for unpaired data, analysis of covariance (ANCOVA) for continuous variables, Kolmogorov–Smirnov test for discretely distributed data, and one-way ANOVA with *post hoc* Tukey–Kramer test for multiple data sets. All the data were collected and analysed using a double-blind approach.

## Additional Information

**How to cite this article**: Shinoda, Y. *et al.* CAPS1 stabilizes the state of readily releasable synaptic vesicles to fusion competence at CA3–CA1 synapses in adult hippocampus. *Sci. Rep.* **6**, 31540; doi: 10.1038/srep31540 (2016).

## Supplementary Material

Supplementary Information

## References

[b1] Sudhof T. C. & Rothman J. E. Membrane fusion: grappling with SNARE and SM proteins. Science 323, 474–477 (2009). 19164740 10.1126/science.1161748 PMC3736821

[b2] Augustin I., Rosenmund C., Sudhof T. C. & Brose N. Munc13-1 is essential for fusion competence of glutamatergic synaptic vesicles. Nature 400, 457–461 (1999). 10440375 10.1038/22768

[b3] Varoqueaux F. *et al.* Total arrest of spontaneous and evoked synaptic transmission but normal synaptogenesis in the absence of Munc13-mediated vesicle priming. Proc. Natl. Acad. Sci. USA 99, 9037–9042 (2002). 12070347 10.1073/pnas.122623799 PMC124419

[b4] Gundelfinger E. D. & Fejtova A. Molecular organization and plasticity of the cytomatrix at the active zone. Curr. Opin. Neurobiol. 22, 423–430 (2012). 22030346 10.1016/j.conb.2011.10.005

[b5] Cisternas F. A., Vincent J. B., Scherer S. W. & Ray P. N. Cloning and characterization of human CADPS and CADPS2, new members of the Ca2+-dependent activator for secretion protein family. Genomics 81, 279–291 (2003). 12659812 10.1016/s0888-7543(02)00040-x

[b6] Speidel D. *et al.* A family of Ca2+-dependent activator proteins for secretion: comparative analysis of structure, expression, localization, and function. J. Biol. Chem. 278, 52802–52809 (2003). 14530279 10.1074/jbc.M304727200

[b7] Walent J. H., Porter B. W. & Martin T. F. A novel 145 kd brain cytosolic protein reconstitutes Ca(2+)-regulated secretion in permeable neuroendocrine cells. Cell 70, 765–775 (1992). 1516133 10.1016/0092-8674(92)90310-9

[b8] Ann K., Kowalchyk J. A., Loyet K. M. & Martin T. F. Novel Ca2+-binding protein (CAPS) related to UNC-31 required for Ca2+-activated exocytosis. J. Biol. Chem. 272, 19637–19640 (1997). 9289490 10.1074/jbc.272.32.19637

[b9] Sadakata T. *et al.* The secretory granule-associated protein CAPS2 regulates neurotrophin release and cell survival. J. Neurosci. 24, 43–52 (2004). 14715936 10.1523/JNEUROSCI.2528-03.2004 PMC6729559

[b10] Berwin B., Floor E. & Martin T. F. CAPS (mammalian UNC-31) protein localizes to membranes involved in dense-core vesicle exocytosis. Neuron 21, 137–145 (1998). 9697858 10.1016/s0896-6273(00)80521-8

[b11] Tandon A. *et al.* Differential regulation of exocytosis by calcium and CAPS in semi-intact synaptosomes. Neuron 21, 147–154 (1998). 9697859 10.1016/s0896-6273(00)80522-x

[b12] Renden R. *et al.* Drosophila CAPS is an essential gene that regulates dense-core vesicle release and synaptic vesicle fusion. Neuron 31, 421–437 (2001). 11516399 10.1016/s0896-6273(01)00382-8

[b13] Sadakata T. *et al.* Differential distributions of the Ca2+-dependent activator protein for secretion family proteins (CAPS2 and CAPS1) in the mouse brain. J. Comp. Neurol. 495, 735–753 (2006). 16506193 10.1002/cne.20947

[b14] Sadakata T. *et al.* Autistic-like phenotypes in Cadps2-knockout mice and aberrant CADPS2 splicing in autistic patients. J. Clin. Invest. 117, 931–943 (2007). 17380209 10.1172/JCI29031 PMC1821065

[b15] Shinoda Y. *et al.* Calcium-dependent activator protein for secretion 2 (CAPS2) promotes BDNF secretion and is critical for the development of GABAergic interneuron network. Proc. Natl. Acad. Sci. USA 108, 373–378 (2011). 21173225 10.1073/pnas.1012220108 PMC3017206

[b16] Sadakata T. *et al.* Reduced axonal localization of a Caps2 splice variant impairs axonal release of BDNF and causes autistic-like behavior in mice. Proc. Natl. Acad. Sci. USA 109, 21104–21109 (2012). 23213205 10.1073/pnas.1210055109 PMC3529019

[b17] Sadakata T. *et al.* Impaired cerebellar development and function in mice lacking CAPS2, a protein involved in neurotrophin release. J. Neurosci. 27, 2472–2482 (2007). 17344385 10.1523/JNEUROSCI.2279-06.2007 PMC6672497

[b18] Jockusch W. J. *et al.* CAPS-1 and CAPS-2 are essential synaptic vesicle priming proteins. Cell 131, 796–808 (2007). 18022372 10.1016/j.cell.2007.11.002

[b19] Fujita Y. *et al.* Ca2+-dependent activator protein for secretion 1 is critical for constitutive and regulated exocytosis but not for loading of transmitters into dense core vesicles. J. Biol. Chem. 282, 21392–21403 (2007). 17540763 10.1074/jbc.M703699200

[b20] Speidel D. *et al.* CAPS1 and CAPS2 regulate stability and recruitment of insulin granules in mouse pancreatic beta cells. Cell Metab. 7, 57–67 (2008). 18177725 10.1016/j.cmet.2007.11.009

[b21] Grishanin R. N. *et al.* CAPS acts at a prefusion step in dense-core vesicle exocytosis as a PIP2 binding protein. Neuron 43, 551–562 (2004). 15312653 10.1016/j.neuron.2004.07.028

[b22] Koch H., Hofmann K. & Brose N. Definition of Munc13-homology-domains and characterization of a novel ubiquitously expressed Munc13 isoform. Biochem. J. 349, 247–253 (2000). 10861235 10.1042/0264-6021:3490247 PMC1221144

[b23] James D. J., Kowalchyk J., Daily N., Petrie M. & Martin T. F. CAPS drives trans-SNARE complex formation and membrane fusion through syntaxin interactions. Proc. Natl. Acad. Sci. USA 106, 17308–17313 (2009). 19805029 10.1073/pnas.0900755106 PMC2765074

[b24] Betz A., Okamoto M., Benseler F. & Brose N. Direct interaction of the rat unc-13 homologue Munc13-1 with the N terminus of syntaxin. J. Biol. Chem. 272, 2520–2526 (1997). 8999968 10.1074/jbc.272.4.2520

[b25] Richmond J. E., Weimer R. M. & Jorgensen E. M. An open form of syntaxin bypasses the requirement for UNC-13 in vesicle priming. Nature 412, 338–341 (2001). 11460165 10.1038/35085583 PMC2585764

[b26] Speidel D. *et al.* CAPS1 regulates catecholamine loading of large dense-core vesicles. Neuron 46, 75–88 (2005). 15820695 10.1016/j.neuron.2005.02.019

[b27] Imig C. *et al.* The morphological and molecular nature of synaptic vesicle priming at presynaptic active zones. Neuron 84, 416–431 (2014). 25374362 10.1016/j.neuron.2014.10.009

[b28] Sadakata T. *et al.* CAPS1 deficiency perturbs dense-core vesicle trafficking and Golgi structure and reduces presynaptic release probability in the mouse brain. J. Neurosci. 33, 17326–17334 (2013). 24174665 10.1523/JNEUROSCI.2777-13.2013 PMC6618362

[b29] Sudhof T. C. Neurotransmitter release: the last millisecond in the life of a synaptic vesicle. Neuron 80, 675–690 (2013). 24183019 10.1016/j.neuron.2013.10.022 PMC3866025

[b30] Becker N., Wierenga C. J., Fonseca R., Bonhoeffer T. & Nagerl U. V. LTD induction causes morphological changes of presynaptic boutons and reduces their contacts with spines. Neuron 60, 590–597 (2008). 19038217 10.1016/j.neuron.2008.09.018

[b31] Yang G. *et al.* Reduced synaptic vesicle density and active zone size in mice lacking amyloid precursor protein (APP) and APP-like protein 2. Neurosci. Lett. 384, 66–71 (2005). 15919150 10.1016/j.neulet.2005.04.040

[b32] Reim K. *et al.* Complexins regulate a late step in Ca2+-dependent neurotransmitter release. Cell 104, 71–81 (2001). 11163241 10.1016/s0092-8674(01)00192-1

[b33] Wu L. G. & Borst J. G. The reduced release probability of releasable vesicles during recovery from short-term synaptic depression. Neuron 23, 821–832 (1999). 10482247 10.1016/s0896-6273(01)80039-8

[b34] Rosenmund C. & Stevens C. F. Definition of the readily releasable pool of vesicles at hippocampal synapses. Neuron 16, 1197–1207 (1996). 8663996 10.1016/s0896-6273(00)80146-4

[b35] Watanabe S. *et al.* Ultrafast endocytosis at mouse hippocampal synapses. Nature 504, 242–247 (2013). 24305055 10.1038/nature12809 PMC3957339

[b36] De Simoni A., Griesinger C. B. & Edwards F. A. Development of rat CA1 neurones in acute versus organotypic slices: role of experience in synaptic morphology and activity. J. Physiol. 550, 135–147 (2003). 12879864 10.1113/jphysiol.2003.039099 PMC2343027

[b37] James D. J. & Martin T. F. CAPS and Munc13: CATCHRs that SNARE Vesicles. Front. Endocrinol. (Lausanne) 4, 187 (2013). 24363652 10.3389/fendo.2013.00187 PMC3849599

[b38] Schoch S. *et al.* Redundant functions of RIM1alpha and RIM2alpha in Ca(2+)-triggered neurotransmitter release. EMBO J. 25, 5852–5863 (2006). 17124501 10.1038/sj.emboj.7601425 PMC1698877

[b39] Kabachinski G., Yamaga M., Kielar-Grevstad D. M., Bruinsma S. & Martin T. F. CAPS and Munc13 utilize distinct PIP2-linked mechanisms to promote vesicle exocytosis. Mol. Biol. Cell 25, 508–521 (2014). 24356451 10.1091/mbc.E12-11-0829 PMC3923642

[b40] Koch M. & Holt M. Coupling exo- and endocytosis: an essential role for PIP(2) at the synapse. Biochim. Biophys. Acta 1821, 1114–1132 (2012). 22387937 10.1016/j.bbalip.2012.02.008

[b41] Wilke S. A. *et al.* Deconstructing complexity: serial block-face electron microscopic analysis of the hippocampal mossy fiber synapse. J. Neurosci. 33, 507–522 (2013). 23303931 10.1523/JNEUROSCI.1600-12.2013 PMC3756657

