# Supplementary material for: CAPS1 stabilizes the state of readily releasable synaptic vesicles to fusion competence at CA3–CA1 synapses in adult hippocampus
Source: Sci Rep. 2016 Aug 22;6:31540. doi: 10.1038/srep31540 (PMC4992871; doi:10.1038/srep31540)

Supplemental Information

CAPS1 stabilizes the state of readily releasable synaptic vesicles to fusion competence at CA3–CA1 synapses in adult hippocampus

Yo Shinoda, Chiaki Ishii, Yugo Fukazawa, Tetsushi Sadakata, Yuki Ishii, Yoshitake Sano, Takuji Iwasato, Shigeyoshi Itohara, and Teiichi Furuichi

Supplemental Methods

Hippocampal primary cell cultures and transfection

Hippocampal primary culture was performed as described previously[1] with partial modification. Primary cultured hippocampal neurons were prepared from *Caps1*$^{flox/flox}$ mice on postnatal day 0. To obtain hippocampal neurons, hippocampi were removed into ice cold modified HBSS (9.5 g/L Hank's balanced salts, 0.8 g/L D-glucose, 1.668 g/L HEPES, 0.35 g/L $NaHCO_3$, pH 7.3; Sigma-Aldrich), digested in papain (45 U/mL; Worthington) for 10 min at 37 °C, and plated onto polyethylenimine (Fluka)-coated glass coverslips (Matsunami) at a density of 2×10^5 mL^{-1} in Neurobasal medium (Invitrogen) supplemented with 2% B-27 (Gibco), 2% Glutamax (Invitrogen) and 1% penicillin/streptomycin (Wako). At 6 days *in vitro*, neurons were transfected with pCAG-EGFP or cotransfected with pCAG-EGFP and pCAG-NLS-Cre using Lipofectamine 2000 (Invitrogen) in antibiotic-free Neurobasal medium. Time-lapse live-cell imaging was performed 2 days after transfection.

Time-lapse live-cell imaging

Time-lapse imaging of SV secretion was performed as described previously[2], with partial modification. FM 4-64 dye was loaded into transfected neurons by incubating them in high-potassium loading buffer ([in mM] 104 NaCl, 47 KCl, 2 $CaCl_2$, 2 $MgCl_2$, 10 D-glucose, 10 HEPES, pH 7.3) containing 10 μM FM 4-64 for 5 min at room temperature. Additional subsequent loading was performed by incubating the cells for 5 min with recording solution ([in mM] 150 NaCl, 4 KCl, 2 $CaCl_2$, 2 $MgCl_2$, 10 D-glucose, 10 HEPES, pH 7.3) containing 10 μM FM 4-64 at room temperature. Excess dye was removed by incubating the cells with recording solution containing 1 μM TTX for 5 min at RT (twice). The coverslip containing the cells was placed into an imaging chamber (Warner Instruments) immersed in non-perfused recording solution containing 1 μM TTX, and the baseline was recorded for 20 s in recording solution containing 1 μM TTX at room temperature. Depolarizing stimulation was carried out by bath application of high-potassium solution by hand ([in mM] 58 NaCl, 90 KCl, 2 $CaCl_2$, 2 $MgCl_2$, 10 D-glucose, 10 HEPES, pH 7.3) containing 1 μM TTX (used as the recording solution in the chamber), to achieve a final potassium concentration of 47 mM. The images were collected using an Eclipse TE2000-E inverted microscope (Nikon) equipped with 60× objective (Nikon) and CoolSNAP HQ2 cooled CCD camera (Photometrics). The excitation filter, beam splitter and emission filter were respectively 470/40, 505 and 536/40 for EGFP, and 540/25, 565 and 605/55 for FM 4-64. Synapses within the field of view were randomly selected for analysis (according to EGFP fluorescence where

applicable). Time-lapse imaging data were acquired and analysed with MetaMorph (Molecular Devices) and Excel (Microsoft) software. All the imaging data were collected from at least 6 coverslips or 3 independent cultures.

Supplemental Text

Acute *Caps1* deletion in individual neurons impairs the release of recycling pool vesicles

We next examined whether developmental defects in *Caps1* cKO mice might affect synapse formation and the maturation of hippocampal neurons, thereby indirectly causing the reduction in synaptic transmission. To test this possibility, we produced an acute deletion of the *Caps1* gene in individual neurons by transfecting primary hippocampal cultures prepared from *Caps1*$^{flox/flox}$ mice with a nuclear localization signal-fused Cre recombinase (NLS-Cre) expression vector together with an EGFP expression vector (the latter used as a marker of transfection and to permit axon tracing). The nuclei of all EGFP-expressing neurons examined were labelled with the anti-Cre antibody, indicating co-expression of NLS-Cre in EGFP-fluorescing neurons (Supplemental Fig. 1A, statistical data not shown). Cells transfected with NLS-Cre and EGFP, but not EGFP alone (control), had low levels of CAPS1 immunoreactivity, indicating that *Caps1* was successfully deleted in these neurons (Supplemental Fig. 1B). Next, we analysed SV release from these neurons with acute *Caps1* deletion. The cells were loaded with a lipophilic styryl *dye,* FM4-64, which is thought to be loaded into recycling SVs, and monitored with time-lapse fluorescence imaging. FM4-64

fluorescent puncta were localized on the axons of untransfected neurons and neurons transfected with either EGFP alone (control) or EGFP and NLS-Cre (acute KO) (Supplemental Fig. 1C, left three panels). Upon depolarizing stimulation (performed with bath application of 47 mM KCl solution), the fluorescence intensity of FM4-64 puncta decreased in all untransfected control, EGFP-transfected and acute KO neurons. The fluorescence intensity declined relatively slow in acute KO neurons than in control neurons (τ = 25.1 ± 0.2 [n = 55], 24.5 ± 0.4 [n = 40] and 27.0 ± 0.3 s [n = 43] for untransfected, EGFP transfected control and acute KO, respectively) (Supplemental Fig. 1D). In addition, the amplitude of the fluorescence change 50 s after KCl application was significantly reduced in acute KO neurons compared with control (untransfected: 63.5 ± 1.79 % [n = 55]; EGFP-transfected control: 60.9 ± 2.36 % [n = 40]; acute KO: 48.0 ± 2.94 % [n = 43]) (Supplemental Fig. 1E). The average intensity of the initial fluorescence was also reduced in acute KO neurons compared with untransfected or EGFP-transfected control neurons (untransfected: 7.41 ± 0.29 [n = 55]; EGFP-transfected control: 7.59 ± 0.53 [n = 40]; acute KO: 4.10 ± 0.37 [n = 43]) (Supplemental Fig. 1F), showing that even the initial exocytotic (and/or recycling) events per synapse were diminished in acute KO neurons. Taken together, these results suggest that CAPS1 is involved in presynaptic neurotransmitter release, because the impaired release of recycling pool SVs in the cKO was mimicked by acute cellular knockout.

Supplemental Figure Legend

Supplemental Figure S1. CAPS1 immunohistochemical staining of hippocampal slices from 1-month-old control and CAPS1 cKO mice.

Supplemental Figure S2. CAPS1 acute deletion reduces SV release. A, Representative image of hippocampal primary cultured neuron from *Caps1*$^{(fl/fl)}$ mice cotransfected with EGFP and NLS-Cre, and immunostained with an anti-Cre antibody (scale bar: 20 µm). **B**, Representative image of EGFP-transfected or EGFP/NLS-Cre-cotransfected neurons (arrows) immunostained with an anti-CAPS1 antibody, and nuclei stained with DAPI (scale bar: 10 µm). **C**, Representative images of FM 4-64 fluorescence in untransfected (arrow head), EGFP-transfected (arrow) and EGFP/NLS-Cre-cotransfected (arrow) neurons, as well as time-lapse images showing the decrease in FM 4-64 fluorescence over time (scale bar: 3 µm). **D**, The time course of relative FM 4-64 fluorescence intensities in untransfected (white circle), EGFP-transfected (gray circle) and *Caps1* KO (black circle) neurons; $\tau = 25.1 \pm 0.2$ (n = 55), 24.5 ± 0.4 (n = 40 synapses) and 27.0 ± 0.3 s (n = 43 synapses) for untransfected, EGFP-transfected and *Caps1* KO neurons, respectively. $P < 0.05$, ANOVA with *post hoc* Tukey–Kramer test. **E**, Averaged amplitude of FM 4-64 fluorescence 50 s after the start of high-potassium stimulation; n = 55, 40 and 43 synapses for untransfected, EGFP-transfected and EGFP/NLS-Cre-transfected neurons, respectively. $^{**}P < 0.01$, ANOVA with *post hoc* Tukey–Kramer test. **F**, Averaged initial fluorescent intensity; n = 55, 40 and 43 synapses for untransfected, EGFP-transfected and

EGFP/NLS-Cre-transfected neurons, respectively. $**P < 0.01$, ANOVA with *post hoc* Tukey–Kramer test.

Supplemental References

1 Shinoda, Y., Tanaka, T., Tominaga-Yoshino, K. & Ogura, A. Persistent synapse loss induced by repetitive LTD in developing rat hippocampal neurons. *PLoS One* **5**, e10390 (2010).

2 Shinoda, Y. *et al.* BDNF enhances spontaneous and activity-dependent neurotransmitter release at excitatory terminals but not at inhibitory terminals in hippocampal neurons. *Front. Synaptic Neurosci.* **6**, 27 (2014).

Supplemental Figure S1

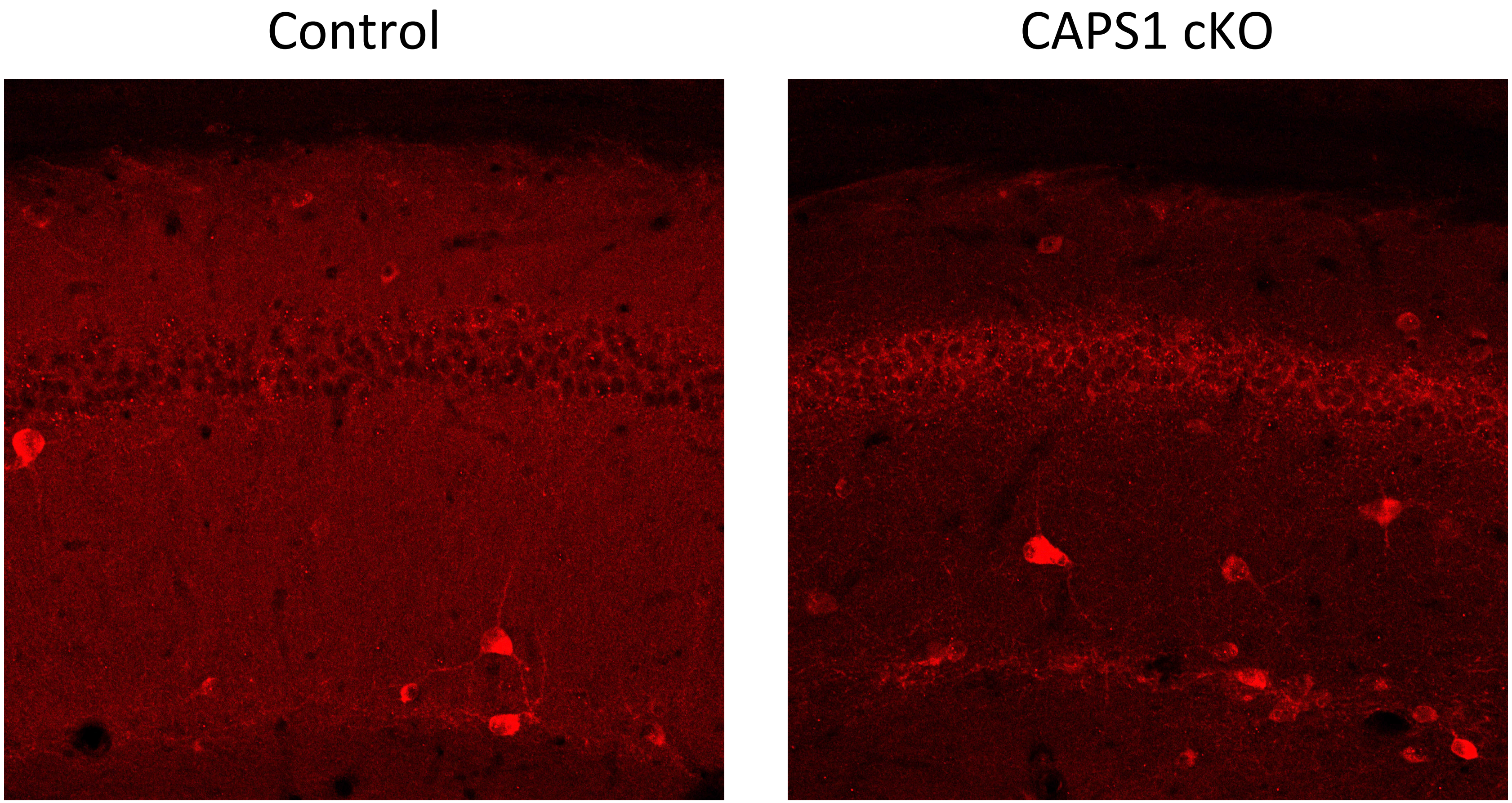

Supplemental Figure S2

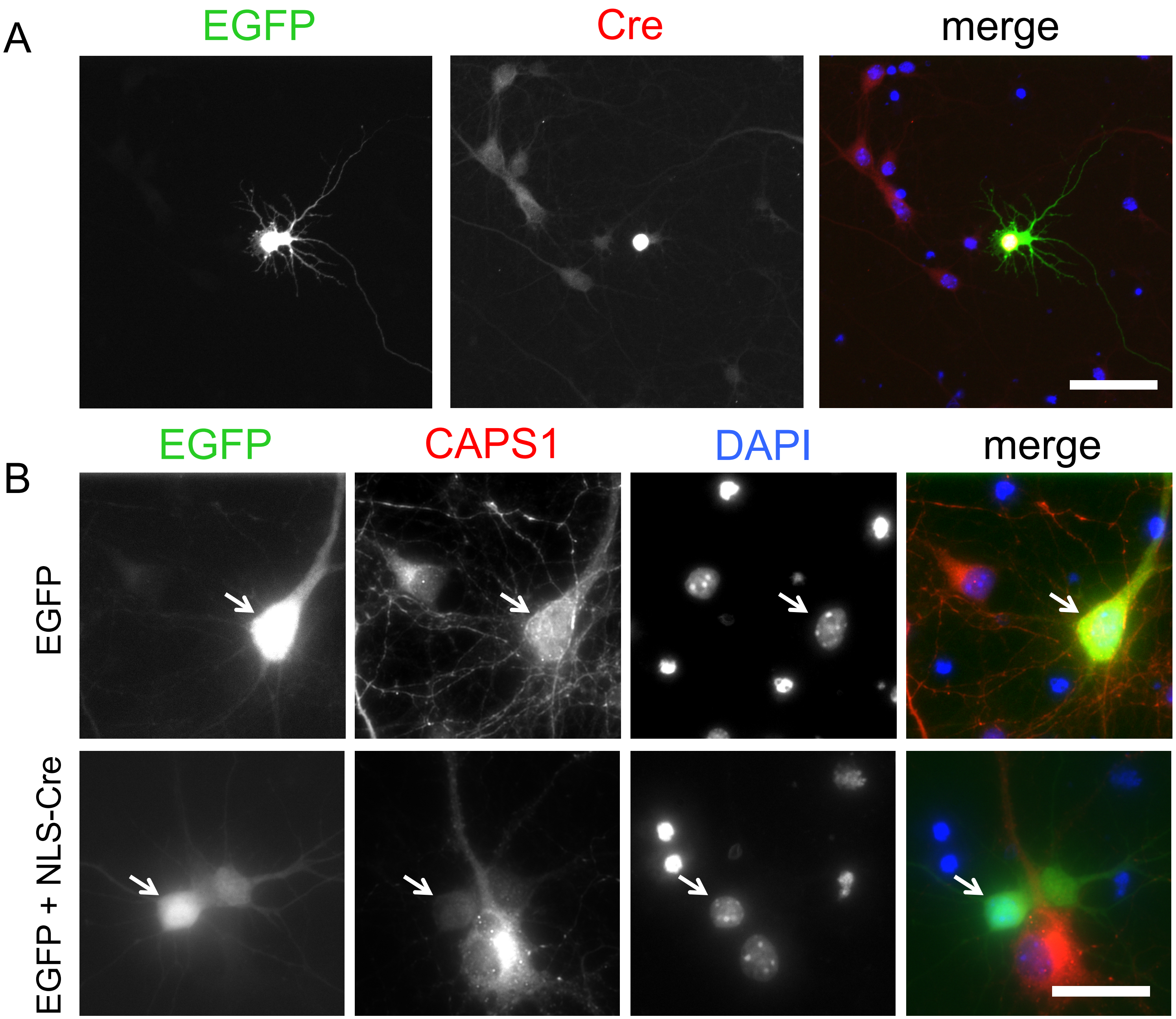

Supplemental Figure S2

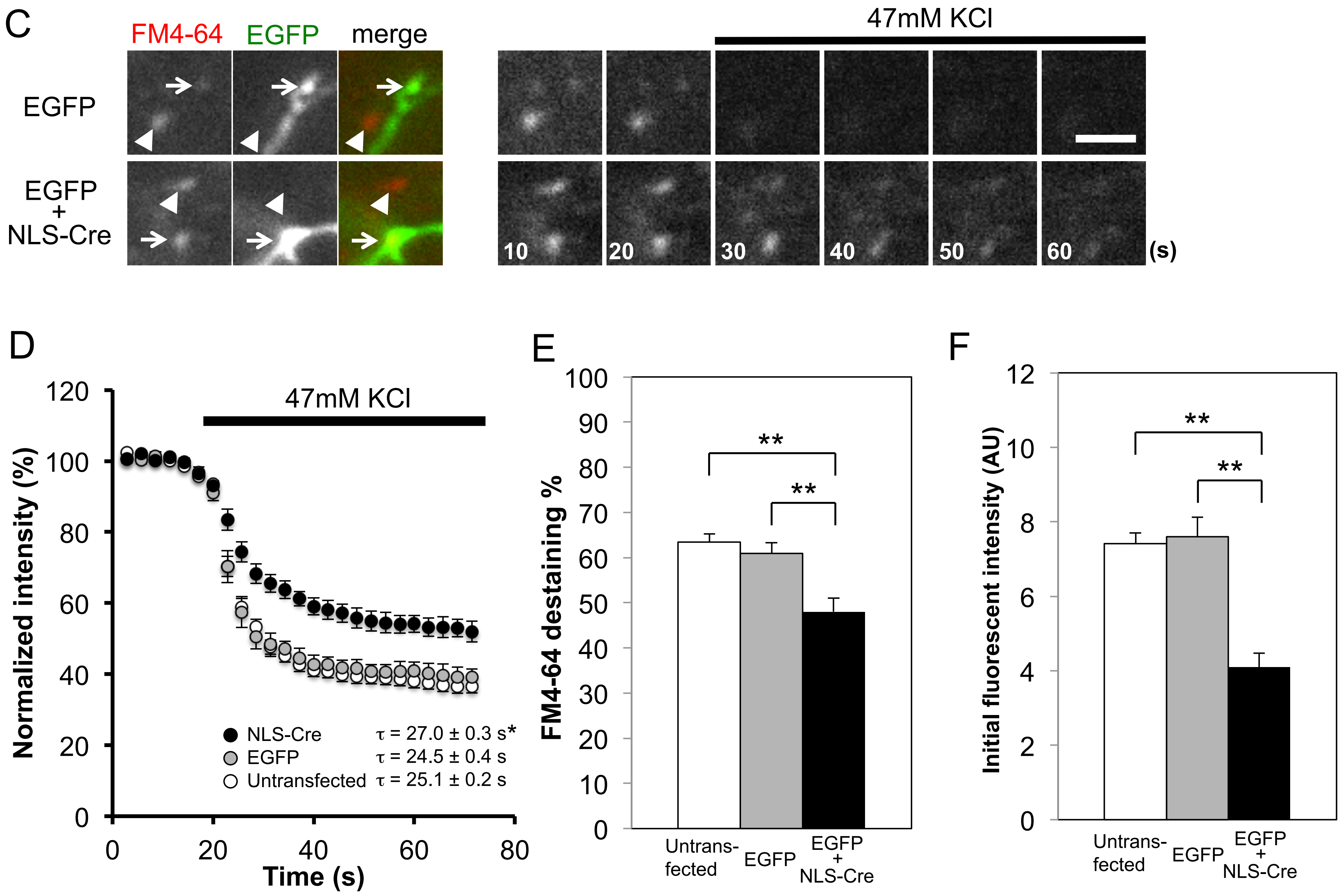

Supplement: Supplementary Information [file srep31540-s1.pdf]
